# One hour of internal precooling with cold water/menthol enhances cycling performance in a heat/wet stress environment: A pilot study

**DOI:** 10.5114/biolsport.2023.116007

**Published:** 2022-07-21

**Authors:** Kévin Rinaldi, Aurélie Collado, Than Tran Trong, Olivier Hue

**Affiliations:** 1 Université des Antilles, ACTES (UPRES EA 3596), UFR STAPS, Pointe-à-Pitre, France; 2 Arkea Samsic Pro cycling team, 35170 Bruz, France; 3 University of Economics – Technology for Industries – Hanoi Vietnam

**Keywords:** Tropical environment, Hot temperature, Humidity, Core temperature, Exercise, Time trial, Cold beverage

## Abstract

The aim of this study was to compare precooling durations before aerobic performance in a heat/ wet stress environment. Seven heat-acclimated and trained male cyclists completed 1-hour time trials in a hot and humid environment. Before each trial, the cyclists drank (1) a neutral beverage at 23°C during the 1-hour pre-exercise resting period (Neutral), (2) an ice-slush/menthol beverage at -1°C during the last 30 min of the resting period (Pre-30), or (3) an ice-slush/menthol beverage at -1°C during the 1-hour pre-exercise resting period (Pre-60). In each condition, the cyclists drank cold water/menthol at 3°C during the exercise. Performance was significantly higher in Pre-60 than in Pre-30 and Neutral conditions (condition effect: F(2,12)=9.50, p=0.003, *η*p^2^=0.61), with no difference between Pre-30 and Neutral. During the resting period, rectal temperature was significantly lower in Pre-60 than in Pre-30 and Neutral (condition effect: F(2,12)=4.48, p=0.035, *η*p^2^=0.43). Thermal comfort and rating of perceived exertion were not affected by conditions, but thermal sensation was positively affected in Pre-60 during the resting period (Friedman condition effect at 40, 45 and 60 minutes: χ^2^=6.74; df=2; p=0.035; χ^2^=8.00; df=2; p=0.018; χ^2^=4.90; df=2; p=0.086, respectively) and exercise (Friedman condition effect at 5 and 60 minutes: χ^2^=6.62; df=2; p=0.037; χ^2^=6.50; df=2; p=0.039, respectively). This study shows that 1 hour of precooling with an ice-slush and menthol beverage (1) improved performance in a 1-hour time trial, (2) had a cumulative effect with a cold water/menthol beverage during this exercise, and (3) decreased rectal temperature during the resting period. This precooling method enhances cycling performance in a heat/wet stress environment.

## INTRODUCTION

A heat/wet stress environment negatively impacts endurance exercise [1]. The processes involved in the alteration of performance have not been fully elucidated and many factors have been advanced to explain this impact (e.g., thermoregulation). Gonzalez-Alonso et al. [2] suggested that the time to exhaustion in hot environments is inversely related to the core body temperature and directly related to the rate of heat storage. Furthermore, the decrease in performance was found to be associated with a higher skin temperature in a warm environment [3]. Other factors include an anticipatory process [4], cardiovascular adjustments [5] and dehydration [3].

Many strategies such as precooling (i.e., before exercise) and percooling protocols (i.e., during exercise) have been used to minimize the impact of the heat/wet stress environment on aerobic performance. Several have been studied, such as cold water immersion [6, 7] and cooling vests [8, 9]. However, it is sometimes difficult to apply these strategies to real sports contexts [1, 10, 11], although internal cooling with beverages is among the easiest to apply [12]. One strategy is to drink cold water/ice slurry or combined cold water/ice slurry and menthol during exercise [10, 13, 14], which was shown to be beneficial for endurance performance in the heat. Indeed, menthol was demonstrated to positively impact thermal sensation and improve performance [12]. Moreover, the authors found that the colder the ingested water is, the higher the performance will be, these results being accentuated when menthol is added to the beverages.

Precooling with cold or iced water has been suggested to enhance performance or time to exhaustion in endurance events [15, 16]. Some have also demonstrated an inverse correlation between (1) the final performance and the increase in core temperature during endurance events (i.e., the greater the increase in core temperature, the higher the performance was [17]) and (2) the final core temperature and the core temperature at the start during long distance exercise (i.e., the lower the core temperature was at the start, the lower it was at the end [18]), both suggesting that beginning with a low core temperature could be useful for events in a heat/wet stress environment. Recently, this hypothesis was tested by Riera et al. [19], who studied the combination of precooling and per-cooling with beverages in a 30-km cycling race in a heat/wet stress environment. The authors reported no effect on performance after 30 min of precooling with a cold beverage (3°C) compared with a control beverage (23°C) on the subsequent 30 km of cycling with per-cooling (ice slush/menthol). They suggested that the volume of beverage drunk during precooling/duration of precooling (i.e., 30 min) was insufficient to induce significant effects on subsequent performance. Furthermore, another study found that a long rest interval after internal precooling by ice ingestion had the greatest benefit for exercise capacity in the heat [20].

Given (1) the apparent insufficiency of 30 min of precooling to improve cycling performance in the heat [19], (2) the positive relation between the rest interval after precooling and performance [20], and (3) a plasma half-life of menthol of 56.2 minutes (for a 100-mg L-menthol capsule) [21], the current study aimed to determine whether the positive results of cold water/menthol ingestion during exercise would be further enhanced by the addition of precooling with increased duration. We hypothesized that a combination of 1-hour internal precooling with ice slurry/menthol ingestion and internal percooling with cold water/menthol would restrict heat stress over the entire exercise period, which in turn would improve performance.

## MATERIALS AND METHODS

Seven heat-acclimated (i.e., living and training in tropical areas) male cyclists (age: 23 ± 5 years, body mass: 70.3 ± 5.7 kg) were recruited from among local cycling teams. Each cyclist was training at least 15 hours per week and had been competing regularly in elite road races. All cyclists completed a medical screening questionnaire and gave written informed consent prior to the study, which was approved by the University Ethics Committee and conducted according to the Declaration of Helsinki.

The cyclists completed three experimental randomized sessions: (1) a control session during which they drank a neutral beverage at 23°C during the 1-hour pre-exercise resting period and cold water/ menthol at 3°C during exercise (Neutral), (2) a session during which they drank an ice-slush/menthol beverage at -1°C during the last 30 min of the resting period and cold water/menthol at 3°C during exercise (Pre-30), and (3) a session during which they drank an iceslush/menthol beverage at -1°C during the 1-hour pre-exercise resting period and cold water/menthol at 3°C during exercise (Pre-60). The experimental sessions were separated by 7 days and were undertaken in a randomized crossover design. The cyclists were instructed to avoid training sessions for 24 hours prior to testing but were allowed 60 min of light-intensity exercise 48 hours before each session. At the start of the session day, the cyclists consumed a standard breakfast that included food and 500 mL of beverage. The sessions began at the same time of day for each session (between 2:00 p.m. and 3:00 p.m.). The experimental sessions were performed in a laboratory reproducing the hot/humid conditions, which were controlled using a portable WBGT meter (HD 32.2, Delta Ohm, Caselle di Selvazzano, Italy; temperature: 30.1 ± 0.5°C; relative humidity, RH: 79 ± 0.3%). During the sessions, the cyclists were not subjected to any flow of ambient air. The L(-)menthol beverage was a 0.025% natural menthol aroma obtained from an 86% menthol-concentrated menthol solution (Robertet, Grasse, France). The ice slurry was produced with an ice slurry machine (Brema, GB 902A, Professional Slush Machine, Ice Makers, Germany). Although ice expands in volume, we carefully ensured that the volume of ice slurry (in mL of water) was precisely the same as the volume of cold water. The temperature of each beverage was measured with a digital thermometer (YSI 409B, Yellow Springs Instruments, OH, USA). A straw with a 1.5-cm diameter was connected to the lid of the water bottle to aid ingestion of the ice slurry.

The cyclists warmed up for 5 min by cycling and performed a 1-hour time trial at the fastest possible speed. Cycling exercises were performed on each participant’s own bicycle fixed on a cycle trainer (Tacx Satori T1856 Tacx BV, Wassenaarn, the Netherlands). The participants’ equipment corresponded to standard cycling equipment. The participants were instructed to keep the same equipment and bicycle for the entire experiment. Throughout the sessions, the cyclists did not benefit from additional motivation. During the 1 hour of pre-exercise (i.e., the resting period), the cyclists were seated and drank 8 g · kg^-1^ of neutral water (Neutral condition) or ice slush (Pre-60 condition) divided into five intakes at 0, 15, 30, 45 and 60 min. During the 30 min of pre-exercise, the cyclists, already seated for 30 min, drank 8 g · kg^-1^ of ice slush (Pre-30 condition) divided into three intakes at 30, 45 and 60 min. During exercise in the three conditions, the cyclists drank 8 g · kg^-1^ of cold water/menthol divided into four intakes at 0, 15, 30 and 45 min of exercise.

Rectal temperature (Trec) was monitored continuously with a rectal probe (YSI409 AC, Yellow Springs Instruments, OH, USA) self-inserted 10 cm beyond the anal sphincter. Heart rate (HR) was continuously monitored using a portable telemetry unit (Suunto Memory Belt, Suunto, Vantaa, Finland) with recording every second. Perceived thermal comfort (TC, from 1: “not uncomfortable” to 4: “very uncomfortable”) and perceived thermal sensation (TS, from 1: “slightly cool” to 7: “extremely hot”) were determined using scales with the range of scores adapted from Hodder and Parsons [22]. Cyclists were asked to rate their perceived exertion according to the grades of Borg’s Rating of Perceived Exertion (RPE) scale [23]. Trec, HR, TC and TS were recorded every 5 min during all the precooling conditions (from 0 to 60 min) and exercise. RPE and performance were recorded every 5 min during exercise. The global performance is given by the total distance covered in 1 hour. The performance was also expressed by 5-min blocks.

Performance and RPE were analysed during exercise, whereas Trec, TS and TC were analysed during the resting and exercise periods. Once the assumption of normality was confirmed (except for TS), parametric tests were performed. To examine our hypotheses regarding the changes in performance, Trec, TC and RPE, one-way (for global performance) or two-way (for performance expressed by 5-min blocks, Trec, TC and RPE) analyses of variances (ANOVA) with repeated measures (i.e., precooling condition × time) were conducted and Tukey’s post-hoc test was assessed when required. For TS, a Friedman ANOVA and a Wilcoxon test were used to assess whether differences existed between conditions and within conditions (i.e., time measures). For technical reasons, the heart rate could not be processed. Data analysis was performed using Statistica 8.0 (StatSoft). Significance was set at p < 0.05. All data are presented as mean ± SD.

## RESULTS

**FIG. 1:**
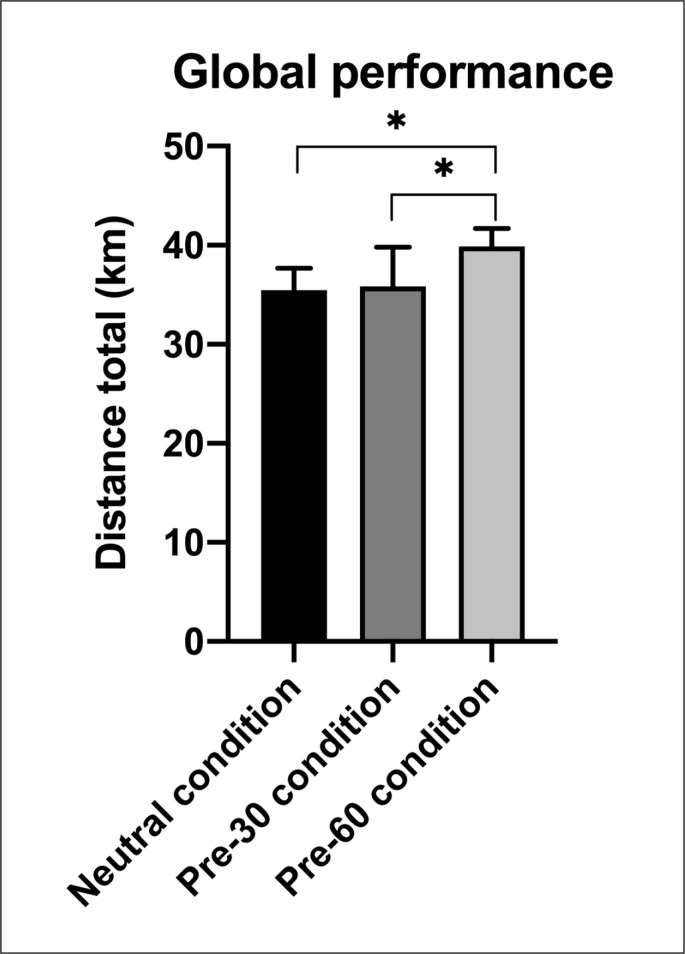
Comparison of global performance between Neutral, Pre-30 and Pre-60 conditions. Significant differences are indicated as follows: *p < 0.01.

**FIG. 2:**
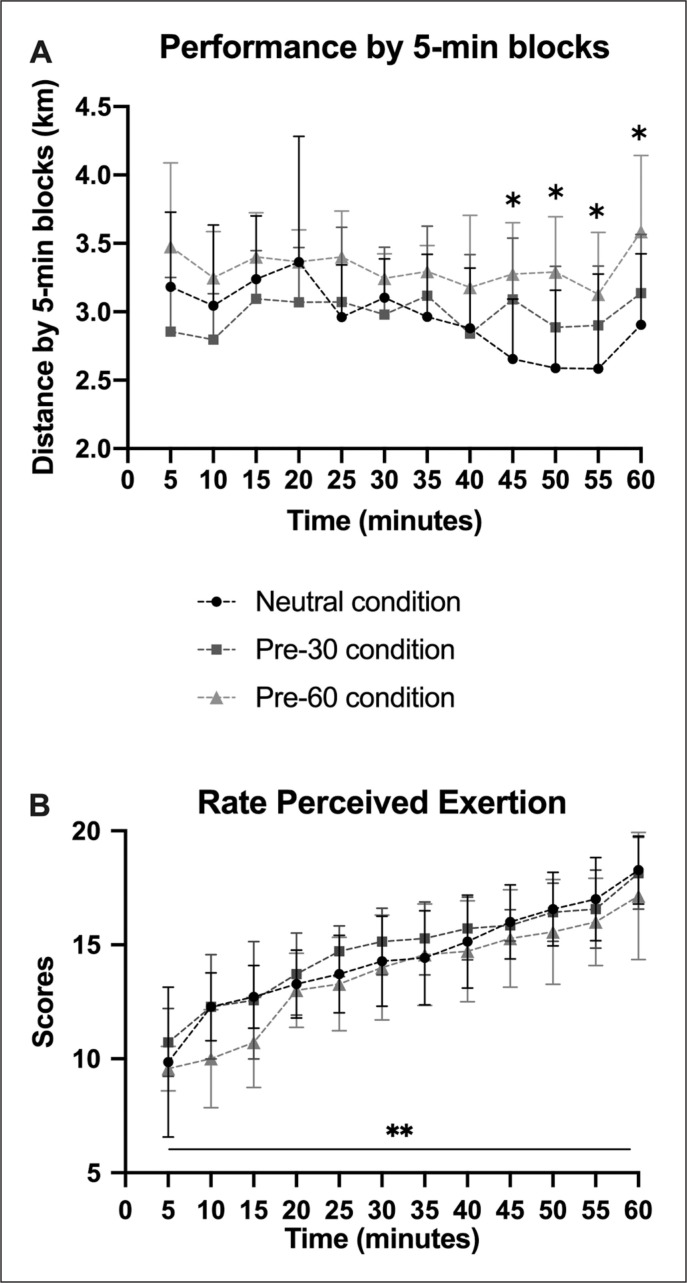
Comparison of performance by 5-min blocks (A) and the rating of perceived exertion (B) between Neutral, Pre-30 and Pre-60 conditions. Significant differences are indicated as follows: *p < 0.01 and **p < 0.001.

Global performance (Figure 1) was affected by condition [F(2,12)=9.50, p=0.003, *ηp*^2^=0.61]: in the Pre-60 condition (39.89 ± 1.81 km), cyclists performed better than in Pre-30 (35.84 ± 3.98 km; p < 0.01) and Neutral (35.47 ± 2.24 km; p < 0.01), and no difference was found between Pre-30 and Neutral. When expressed by 5-min blocks (Figure 2A), performance in Pre-60 (3.32 ± 0.4 km) was higher than in Pre-30 (2.99 ± 0.4 km; p < 0.01) and Neutral (2.96 ± 0.6 km; p < 0.01). Significant differences occurred from the 9^th^ block (i.e., 45 min), with Pre-60 higher than Pre-30 and Neutral. However, performance was not affected by time. The perception of exertion (Figure 2B) increased over time [F(11,66)=33.72, p < 0.001, *ηp*^2^=0.85], but no differences were observed for condition [F(2,12)=2.01, ns, *ηp*^2^=0.23] and condition × time [F(22,132)=1.09, ns, *ηp*^2^=0.15].

**FIG. 3:**
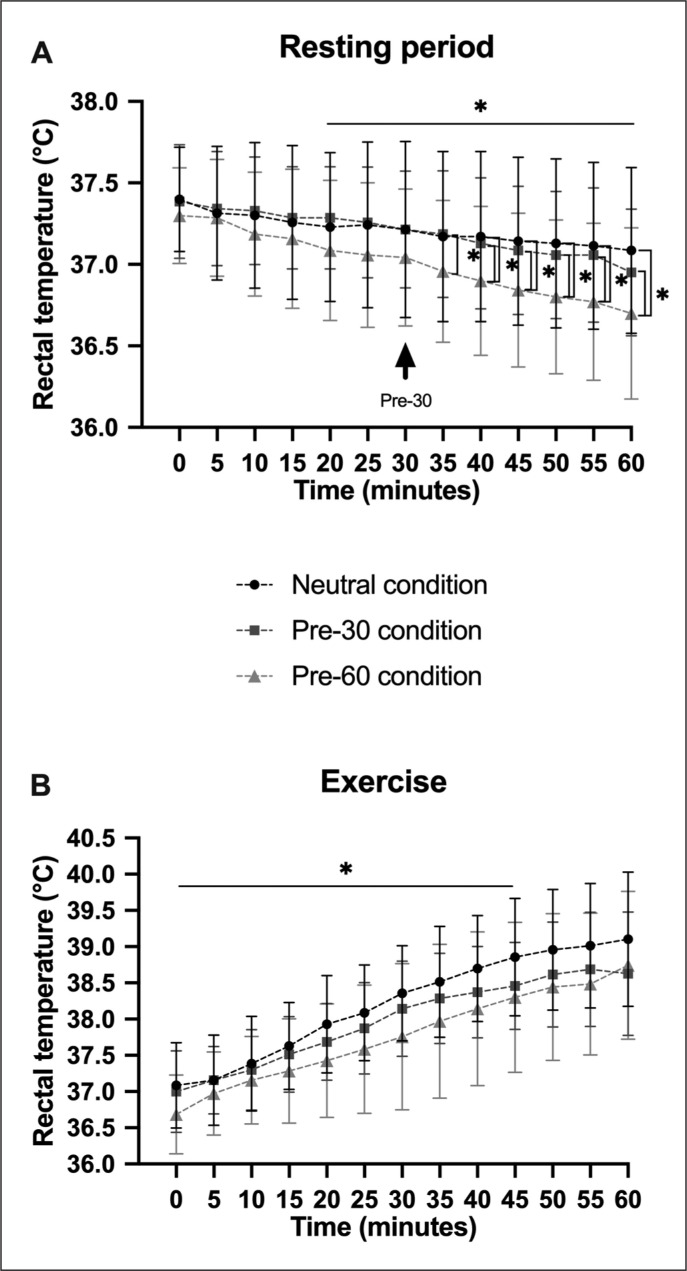
Comparison of rectal temperature during resting period (A) and exercise (B) between Neutral, Pre-30 and Pre-60 conditions. Significant differences are indicated as follows: *p < 0.05.

During the resting period, the mean Trec (Figure 3A) was affected by condition [F(2,12)=4.48, p=0.035, *ηp*^2^=0.43]; by time [F(12,72)=25.30, p < 0.001, *ηp*^2^=0.81], with a decrease during the resting period at each time from the 20^th^ minute (p < 0.05); and by condition × time [F(24,144)=1.97, p=0.008, *ηp*^2^=0.25]. Tukey’s post-hoc analysis indicated that the mean Trec was lower in the Pre-60 condition compared with the Neutral (p=0.049) and Pre-30 conditions (p=0.069) from the 40^th^ (T40: p=0.002; from T45 to T60: p < 0.001) and the 35^th^ minute (T35 and T40: p=0.04; T45: p=0.016; T50: p=0.006; T55: p < 0.001; T60: p=0.01). During exercise (Figure 3B), mean Trec increased over time [F(12,72)=81.90, p < 0.001, *ηp*^2^=0.93]. The post-hoc test showed that mean Trec increased at each time (p < 0.05) up to 40 min, with no difference for the last four blocks (T45, T50, T55 and T60). No differences were found for condition [F(2,12)=1.62, ns, *ηp*^2^=0.21] or condition × time [F(24,144)=1.21, ns, *ηp*^2^=0.17].

During the resting period, TC was not different between conditions [F(2,12)=0.03, ns, *ηp*^2^=0.00], time [F(12,72)=0.78, ns, *ηp*^2^=0.12] or condition × time [F(24,144)=0.63, ns, *ηp*^2^=0.10] (data not shown). During exercise, only TC increased over time [F(12,72)=3.84, p < 0.001, *ηp*^2^=0.39], but no differences were observed for condition [F(2,12)=0.31, ns, *ηp*^2^=0.05] and condition × time [F(24,144)=0.44, ns, *ηp*^2^=0.07] (data not shown). Regarding TS during the resting period, a Friedman ANOVA indicated a decrease between times measures from 0 to 60 min for the Pre-30 condition (Friedman χ^2^=29.86; df=12; p=0.003) and the Pre-60 condition (Friedman χ^2^=38.94; df=12; p < 0.001). Significant differences were observed between conditions at 40 min (Friedman χ^2^=6.74; df=2; p=0.035) and 45 min (Friedman χ^2^=8.00; df=2; p=0.018), but the difference was not significant for 60 min (Friedman χ^2^=4.90; df=2; p=0.086). TS was significantly lower in the Pre-60 condition compared with the Neutral condition at 40 min (T=0, Z=2.02, p < 0.05) and 45 min (T=0, Z=2.02, p < 0.05), but the lower score in the Pre-60 condition compared with the Neutral condition was not significant (T=0, Z=1.83, p=0.068) (data not shown). During exercise, a significant increase between time measures from 0 to 60 min was observed for the Pre-30 condition (Friedman χ^2^=36.52; df=12; p < 0.001), but it was not significant for the Pre-60 condition (Friedman χ^2^=20.47; df=12; p=0.059). Differences were observed between conditions at 5 min (Friedman χ^2^=6.62; df=2; p=0.037) and 60 min (Friedman χ^2^=6.50; df=2; p=0.039). Compared with the Neutral condition, TS in the Pre-60 condition scored lower at 5 min, although not significantly (T=0, Z=1.83, p=0.068), and was significantly lower at 60 min (T=0, Z=2.02, p < 0.05) (data not shown).

## DISCUSSION

The aim of this study was to determine whether a combination of 1-hour internal precooling with ice slurry/menthol ingestion and internal per-cooling with cold water/menthol would restrict heat stress over the entire exercise period and improve performance. The most important results were the following: (1) the combination of 1 hour of internal precooling with ice slush/menthol at -1°C and internal per-cooling with cold water/menthol improved performance during the subsequent 1-hour exercise when compared with both the Pre-30 and Neutral conditions, and (2) during the resting period, rectal temperature was significantly lower in Pre-60 than in Pre-30 and Neutral conditions.

Precooling for 60 min significantly increased performance compared with Pre-30 and Neutral conditions (+4.05 ± 3.81 km; +4.42 ± 1.29 km, respectively), with no difference between the latter two. Whereas Riera et al. [19] examined precooling durations and suggested that longer precooling periods (i.e., > 30 min) might improve subsequent performances, others focused on the effect of length of time after the precooling [20]. Indeed, Naito et al. [20] found that a long rest interval after internal precooling by ice ingestion had the greatest positive effect on exercise capacity in a hot and humid environment. The authors assumed that core temperature (i.e., rectal temperature) and heat storage would be reduced before the start of exercise. Although our protocol differs from theirs, the present Pre-60 condition, with the start of precooling ingestion at least 60 min before the start of the effort, may have been a long enough rest interval to allow the core temperature to drop. This was corroborated by the mean rectal temperature (a good reflection of core temperature), which was lower in the Pre-60 condition than in Pre-30 and Neutral conditions. This reduction in core temperature induced by 60 min of precooling led to a decrease in heat storage, which might have prolonged the time to reach critical temperatures during exercise. As Hasegawa et al. [24] suggested that a decrease of 0.3°C in core temperature could lead to an increase in aerobic performance in a hot and humid environment, the present 0.6°C reduction induced by the ice slurry in the Pre-60 condition may have favoured the improvement in cycling performance. In addition, it was found that the absorption of ice slush reduced body temperature more than the absorption of the same quantity of cold water [11]. The temperature of the ingested beverage could thus explain the difference observed between the two 60-min precooling conditions (Pre-60 and Neutral conditions).

Despite the low temperature of the ingested solution [12–14, 20, 25, 26] and the additive effect of menthol with cold beverage [12], our results did not show a significant difference between the Pre-30 condition (i.e., ice-slush/menthol beverage at -1°C during the last 30 min of the resting period) and Neutral condition (i.e., neutral beverage at 23°C during the 1-hour pre-exercise resting period) for performance or rectal temperature. In accordance with other studies [19, 27], these results seem to confirm that 30 min of precooling is not enough to improve cycling performance.

During the time trial, the increase in Trec was due to the metabolic heat production generated by the exercising muscles. However, we assumed that the limited evacuation process due to the heat/ wet stress environment would induce a similar increase in Trec independently of condition. Regarding performance, Neutral was lower than Pre-60, despite ingestion of cold water/menthol during exercise for both conditions. When expressed by blocks, performance was significantly affected starting from 45 min: from this point onwards, Neutral performance was negatively affected by the heat/wet stress environment, and this result was expected even in natives of tropical climate (i.e., a hot and humid environment) [28]. Pre-60 performance was not as negatively impacted by the environment as Pre-30 and Neutral, and thus it would seem that precooling for 1 hour with ice slush/menthol (Pre-60) enhanced the effect of per-cooling by decreasing thermal stress and heat storage. González-Alonso et al. [2] suggested that the time to exhaustion (i.e., cycling performance until volitional exhaustion) in hot environments is inversely related to the initial temperature and directly related to the rate of heat storage. This argument was corroborated by the significant decrease in Trec for Pre-60 compared with the other pre-exercise conditions.

The increase in performance noted in Pre-60 may also have been linked to the use of menthol. Indeed, as the plasma half-life of menthol is 56.2 minutes for a 100-mg L-menthol capsule [21], Pre-60 might have resulted in an increased concentration of menthol in the brain compared with Pre-30. Menthol could thus act as a stimulant on the central nervous system by inhibiting the recapture of dopamine and/or by facilitating its release [29, 30]. This stimulation enhances ambulatory activity in rats [29, 30]. Menthol is considered effective in the treatment of mental fatigue and may possess an action similar to that of psychostimulants [30–32] by enhancing the intensity of the afferent signals induced by cold water/menthol ingestion during exercise. These processes partly corroborate the central regulator model in the brain introduced by St Clair Gibson and Noakes [33].

Moreover, the absence of a Trec difference between Pre-30 and Neutral conditions at the end of the precooling period might be ascribed to the menthol effect, which limits thermoregulation [34]. The cooler effect of menthol on TRPM8 and TRPA1 could affect thermo-regulation [35] and explain the smaller decrease in Trec in Pre-30 than Pre-60: menthol would block the thermoregulation while the greater amount of ice ingested in Pre-60 would outperform the effects of menthol. However, we assumed that these effects were marginal and the low menthol concentration used in our study partially explained this result. Nevertheless, we also assumed that the main factor to explain the lack of decrease in Trec was the quantity of ice slush given in the 30-min period. The absorption of large quantities of a cold drink (or ice slush) results in greater core temperature reduction than the ingestion of smaller quantities of water at the same temperature. Ross et al. [36] found that ingestion of 500 g and 1000 g of ice slush reduced Trec by 0.25°C and 0.60°C, respectively. In our study, we used 8 g · kg^-1^ (corresponding to 562.2 ± 45.6 g) of ice slush/menthol for 30 min without decreasing Trec. Yet, the absorption of the ice slush/menthol over a longer period significantly decreased Trec (i.e., Pre-60). Yeo et al. [25] found a significant decrease in gastrointestinal temperature after ingestion of 8 g · kg^-1^ over 30 min compared with the ingestion of an ambient beverage (30.9°C) in a heat/wet stress environment. We had some concerns in comparing the ambient beverage used in their study (30.9°C) to our Neutral condition with water at 23°C. Indeed, we could not conclude as to whether the lack of a reduction in Trec was due to the delta between ice slush and neutral water or the effect of menthol.

No differences between conditions were found for TC during the precooling period. These results, in accordance with those of Riera et al. [19], suggest that hydration induced by neutral water or ice slush/menthol did not influence the feeling of comfort in acclimated participants. TS was significantly affected by time during the rest period and decreased at the end of the precooling with ice slush (i.e., Pre-30 and Pre-60 conditions). In 2012, Yeo et al. [25] demonstrated that precooling with 8 g · kg^-1^ of cold drink for 30 min in a hot and wet environment induced a significant decrease in thermal sensation. In our study, the observation that TS was positively affected by time in precooling with ice slush seems to confirm that a precooling hydration protocol with a very low temperature (e.g., ice slush/ menthol for 30 or 60 min) would be positive for subjective perception, such as the thermal sensation in a heat/wet stress environment. This was corroborated by the between-condition differences observed at the end of the resting period, with a lower score on TS in Pre-60 compared with the Neutral condition. During exercise, TC and TS increased significantly over time, which is an obvious consequence of exercising in a tropical environment.

Although no between-condition differences were observed in TC, we found some in TS at exercise initiation (i.e., 5 min) and after exercise (i.e., 60 min). The results obtained at the end of the resting period, with (1) a decrease of the TS score in the Pre-60 condition and (2) a lower score in Pre-60 than the Neutral condition, would explain the lower score in the Pre-60 condition compared with the Neutral condition at the beginning of the effort, thus emphasizing a shortlasting effect. Although this effect disappeared during the effort, probably because of exercise-induced hyperthermia, it is interesting to note that this difference seemed to reappear at the end of the effort, at 60 min, although the difference was not statistically significant. The cyclists seem to have perceived less heat sensation at the end of exercise when they had benefited from 1 hour of internal pre-cooling with cold water/menthol. Last, as TS was positively affected by time during the rest period in this condition, it is possible that the 1 hour of internal precooling helped limit the increase in TS during the cycling performance and thus contributed to enhancing it.

Despite the higher performance in Pre-60, RPE was affected by time but not by condition. These results suggest that the difficulty perceived by the cyclists increased similarly whatever the precooling conditions. As studies failed to show improved performance with 30 min of precooling [19] and no difference was found in our study between Pre-30 and Neutral, we assume that the higher performance in Pre-60 was due to the effect of 1 hour of ice-slush precooling with menthol, which biases the central nervous system by increasing performance without affecting the RPE. Indeed, as menthol is known to exhibit analgesic properties through peripheral and central mechanisms [37], the combination of ice slush and menthol during Pre-60 may have had an analgesic effect, allowing the cyclists to perform better without impacting RPE.

## CONCLUSIONS

This study showed that 1 hour of precooling with an ice-slush and menthol beverage (1) improved cycling performance in a 1-hour time-trial, (2) had a cumulative effect with a cold water/menthol beverage during exercise, and (3) decreased rectal temperature during the pre-exercise resting period. The decrease in the rectal temperature during the 1-hour precooling period could explain the higher performance in the Pre-60 condition, but the cumulative effect of menthol on pre- and per-cooling might have enhanced the intensity of afferent signals within the central nervous system. As this is an exploratory study, caution is needed in interpreting the results, given the small samples. These results will have to be confirmed in future studies with larger samples. In addition, further data are needed to determine the efficiency of this method for other exercise durations. Finally, future studies should also focus on the possibility of reducing the precooling time by combining several strategies, such as internal with external.
